# The Book World of Medicine and Science

**Published:** 1905-10-21

**Authors:** 


### The Book World of Medicine and Science.

Heating by Hot Water, Ventilation, and Hot Water Supply. Third Edition. By Walter Jones, M.I.Mech.E. and M.I.H.V.E. (London: Crosby, Lockwood. 1904. 344 pages. 142 illustrations. 6s.)

This is one of the books that engineers so much like to get hold of. It is written by a man who has practised what he writes about for thirty years and who has also carefully studied the principles of the sciences upon which the work must be based. It is full of information that will be useful to everyone who has anything to do with heating apparatus for any requirement whatever. Descriptions are given of the different methods of distributing heat for the various purposes dealt with, numerous tables are given, together with the results of a great many experiments the author has made himself, some of which go to upset the conclusions arrived at by the great French *savants* who have investigated the matter. French experimenters are usually very careful and therefore very accurate, but it is exceedingly difficult to exactly reproduce in the laboratory all the conditions that are met with in engineering practice, and hence there has grown up in every branch of engineering two schools, the theorists, and the practical men; while there has also arisen the strange paradox that the practical men are often more correct in their theory than the pure theorists, because they have usually more accurately measured all the conditions. Mr. Jones gives sectional and other drawings of every kind of boiler, and the principal forms of calorifier that are used in the different methods of heating, also diagrams showing the arrangements of the pipes for conveying steam and hot water to the rooms to be warmed, or to be supplied with hot water, that will enable anyone with a little engineering knowledge to master this too often intricate problem.

The Needs of Man. A Book of Suggestions. By W. Winslow Hall, M.D. (London: Swan Sonnenschein and Co. Pp. 204. Price 3s. 6d.)

When the bodily, mental, moral, and spiritual needs of mankind have to be compressed into the space here allotted to them, it need cause no surprise that aspirations after ideals, which to ordinary mortals seem outside the bounds of practical politics, jostle the truisms of the commonplace, even with, at times, a suggestion of anti-climax. But, as its author states, the book is suggestive rather than conclusive, and it may be that the reader may derive equal benefit from the contemplation of the far-off vision and from the actualities of daily life. With the teaching of the book when it is positive there is much to agree, and the verses that follow each chapter, if somewhat unconventional in form, point the same moral in a briefer and more tuneful manner.

Hygiene and Public Health. By B. Arthur Whitelegge, C.B., M.D., B.Sc.(Lond.), F.R.C.P., D.P.H., and George Newman, M.D., D.P.H., F.R.S.E. New edition. Illustrated. Pp. 636. 1905. (London: Cassell and Co. Price 7s. 6d.)

Since its first appearance in 1890 this volume has enjoyed the reputation of being one of the best-known of Messrs. Cassell's student's manuals, and the new edition has been thoroughly revised and brought up to date. Though considerable additions have been required, in view of recent legislation and advances of sanitary science, the information is still retained within a moderate compass and the book remains one of the most concise publications on the subject. The tenth edition will well maintain the esteem in which the volume is held by those who have had occasion to use it.

A Manual of Chemistry: Inorganic and Organic. By Arthur P. Luff, M.D., B.Sc.(Lond.), F.R.C.P., F.I.C., and Frederic James M. Page, B.Sc.(Lond.), F.I.C. Third edition. Pp. 555, with 43 illustrations. 1905. (London: Cassell and Co. Price 7s. 6d.)

This handbook is intended for the use of students of medicine, and the new edition has been revised to bring it into conformity with the most recent regulations of the Conjoint Board and of the Society of Apothecaries. There has also been added a short but clearly expressed account of the principles and practice of volumetric analysis, and a section on the chemical composition of the more complex organic substances which have recently been so largely used as remedies.

The Surgical Assistant. By Walter M. Brickner, B.S., M.D. (New York: International Journal of Surgery Company. 1905. Pp. 363. With 123 illustrations. Price $2.00 net.)

It may be said that at least four trained individuals are necessary for the performance of a surgical operation of any considerable magnitude. These will be the surgeon, his assistant, the anæsthetist, and the nurse. Further, it may be stated as a general proposition that for the success of any operative procedure it is absolutely necessary that each of these individuals shall be experienced and well trained in the duties which fall to his share. But while the surgeon, the anæsthetist, and the nurse have an abundance of technical literature at their disposal for reference, it appears that through some curious oversight not even one satisfactory and sufficient text-book appears to have been produced hitherto for the guidance of the surgeon's assistant. Dr. Brickner's attempt to fill up this hiatus with the manual now before us is therefore welcome on account of its extrinsic as well as its intrinsic merits. The book gives much useful information on the duties of hospital internes, preparation for operation, administration of anæsthetics, assistance during special operations, after-treatment of surgical cases, and so forth. At the end of the book is an appendix containing illustrations of surgical instruments in common use. We think Dr. Brickner's text-book will be found of great service to dressers, house-surgeons, general practitioners, and others who may be called upon to assist at surgical operations.

Nelson's Sixpenny Classics. (London: Thomas Nelson and Sons.)

We have received a copy of "Adam Bede" as produced by Messrs. Nelson and Sons in their Sixpenny Classics Series. The book is neatly bound, and, what is much more important, the type is large and clear and the paper used is opaque, so that there is no undue strain thrown upon the eyes of the reader. We always gladly welcome cheap editions of good books such as this, provided that the low cost is not acquired at the expense of the public eyesight.

